# Complicated Spondylodiscitis Caused by Pseudomonas aeruginosa in a Healthy Teenager: A Case Report and Literature Review

**DOI:** 10.7759/cureus.101312

**Published:** 2026-01-11

**Authors:** Mariana Reis, Joana Capela, Inês Almeida, José Gonçalo Marques, Maria João Virtuoso

**Affiliations:** 1 Pediatrics, Unidade Local de Saúde (ULS) Algarve, Faro, PRT; 2 Pediatrics, Unidade de Infeciologia e Imunodeficiências, Unidade Local de Saúde (ULS) Santa Maria, Lisbon, PRT

**Keywords:** antibiotic therapy, ct-guided biopsy, paravertebral abscess, pseudomonas aeruginosa, spondylodiscitis

## Abstract

Pediatric spondylodiscitis (PSD) is a rare but potentially serious infection of the intervertebral disc and adjacent vertebral endplates. Its insidious onset and nonspecific presentation often result in delayed diagnosis, increasing the risk of complications such as vertebral destruction, abscess formation, spinal deformity, and neurological deficits. While *Staphylococcus aureus* and *Kingella kingae* are the most commonly reported pathogens, *Pseudomonas aeruginosa* is exceedingly rare in otherwise healthy children. We report a previously healthy 16-year-old male presenting with persistent lower back pain, intermittent fever, and gait disturbance. MRI revealed D12-L1 spondylodiscitis with bilateral psoas abscesses. Despite broad-spectrum empirical antibiotics, his condition worsened, and blood cultures remained negative. Thirty-two days after admission, a CT-guided abscess biopsy identified *P. aeruginosa*, enabling targeted therapy with piperacillin-tazobactam and aminoglycosides, which led to rapid clinical improvement. Due to progressive thoracolumbar kyphosis, percutaneous posterior spinal stabilization (D10-L2) was performed. The patient completed a six-week antibiotic regimen and physiotherapy, achieving full recovery without residual disability. PSD is challenging to diagnose because of its nonspecific clinical presentation, with reported diagnostic delays averaging four to five weeks. Culture-negative infections are frequent, complicating empirical treatment. *P. aeruginosa* is an exceptionally rare pathogen in PSD, with only two previous cases reported, typically associated with immunosuppression, prior surgery, or contiguous spread. This case highlights the critical role of early imaging and high clinical suspicion in adolescents with persistent back pain unresponsive to standard therapy. Image-guided biopsy of paravertebral or psoas abscesses significantly increases pathogen detection, facilitating effective targeted antimicrobial therapy. Surgical stabilization is warranted in cases of progressive vertebral destruction or spinal instability. Multidisciplinary management is essential for optimal outcomes. Early recognition of PSD, prompt imaging, and targeted biopsy when empirical therapy fails are key to accurate pathogen identification. Timely, pathogen-specific antimicrobial therapy, combined with surgical intervention when indicated, can prevent long-term complications and ensure full recovery in affected children.

## Introduction

Spondylodiscitis (SD) is a rare but potentially serious inflammatory and infectious disease of the spine, involving the intervertebral disc space and adjacent vertebral endplates. It encompasses both discitisand vertebral osteomyelitis, now considered stages of the same pathological process that can, in severe cases, progress to epidural or paraspinal abscess formation [1,2].

Spinal infections may arise from hematogenous spread, direct inoculation following trauma or surgical procedures, or contiguous extension from adjacent tissues, with arterial hematogenous dissemination being the most common route due to the rich vascular supply of the vertebral bodies [2].

SD is uncommon in children, with an estimated incidence of approximately 1:250,000, corresponding to about 2-4% of infectious bone diseases [2], markedly lower than in adults. However, the incidence has been increasing in recent years, partially due to improved diagnostic techniques [3,4].

Clinically, pediatric spondylodiscitis (PSD) often presents insidiously, with symptoms such as refusal to walk or sit, limping, irritability, and unexplained fever [4]. Back pain is a prominent feature, especially in older children, but the nonspecific presentation frequently leads to significant delays in diagnosis. The lumbar and lumbosacral regions of the spine are more frequently involved [5].

MRI is the diagnostic gold standard, while inflammatory markers such as erythrocyte sedimentation rate (ESR) and C-reactive protein (CRP) are typically mildly to moderately elevated [2,4]. Blood cultures frequently yield negative results (positive in less than 30% of patients) [2]. When an organism is identified, the most common causative agent is *Staphylococcus aureus*, followed by *Kingella kingae* in young children and occasionally *Mycobacterium tuberculosis*, particularly in endemic areas [4].

Initial empiric antibiotic therapy for PSD is usually directed against *S. aureus*, with guidelines and reviews recommending broad-spectrum intravenous coverage that includes an agent active against *S. aureus *(and methicillin-resistant* S. aureus *(MRSA)where locally prevalent) [6]; however, clear, evidence-based treatment guidelines for the pediatric population are lacking [1,4].

Early recognition and prompt initiation of antibiotic therapy are critical to prevent long-term complications, including spinal deformity, chronic pain, and neurological deficits [2]. Most cases respond well to conservative management, although surgical intervention is indicated in the presence of abscesses, neurological compromise, or spinal instability [1,3].

We present a case of SD complicated with bilateral psoas abscesses in a healthy teenager.

## Case presentation

A previously healthy 16-year-old male experienced sudden, severe pain in both lower back regions following a high-impact sporting activity (running). It was initially interpreted as traumatic; however, there was no improvement with conservative treatment, including analgesics and muscle relaxants.

One month after the pain onset, he developed intermittent fevers peaking at 39.2°C, predominantly in the evenings, with no associated night sweats or weight loss. He denied any recent respiratory infections, cough, any trauma, or ingestion of any unpasteurized dairy products. The patient reported frequently drinking water from a canteen that he described as being "moldy." He denied being sexually active. He practiced swimming indoors in a heated pool.

Two weeks later, due to persistent symptoms, he was referred to the emergency department (ED) for etiological investigation. Upon admission to the ED, the patient appeared distressed and had difficulty walking. Physical examination revealed tenderness over the spinous processes of D12-L2; the neurological examination was unremarkable. Laboratory evaluation on admission demonstrated elevated CRP (89 mg/L, normal range <5 mg/L) and ESR (61 mm/h, normal range <20 mm/h), and leucocytes of 11,500 leuc/mm³ (normal range 4,000-10,000 leuc/mm³).

An MRI scan of the lumbar spine suggested SD at D12-L1, with an adjacent paravertebral abscess involving the psoas muscle bilaterally. The patient was admitted to the pediatric ward and started on an intravenous course of empiric flucloxacillin and ceftriaxone.

**Figure 1:**
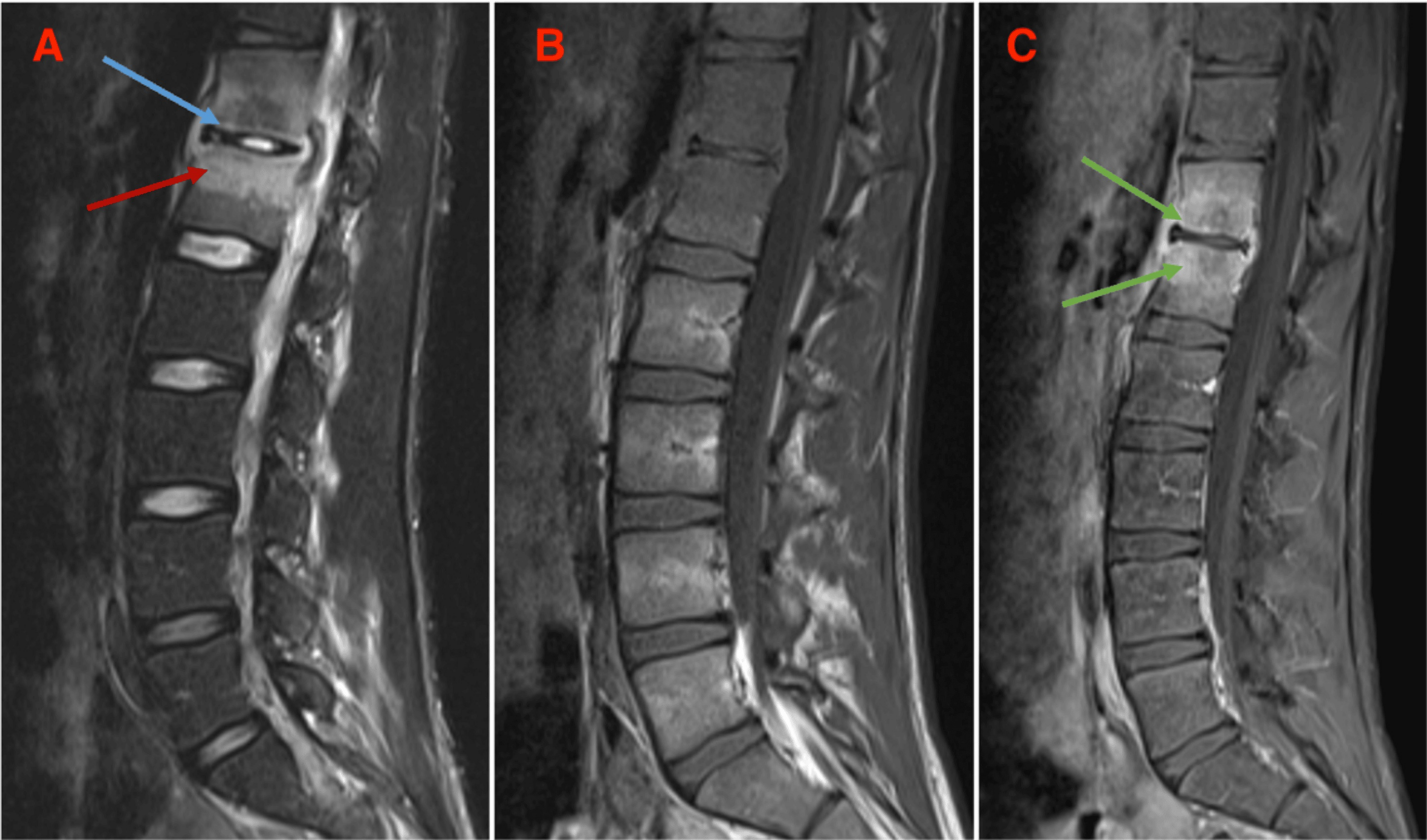
Sagittal T2 STIR sequence (Image A): hyperintensity on the subchondral aspect of the D12–L1 disc (blue arrow) with hyperintensity of the vertebral bodies (red arrow). T1 FS sequence without contrast (Image B) and T1 FS with contrast (Image C): enhancement of the disc and vertebral bodies (green arrow). STIR: short-tau inversion recovery

Despite treatment, he showed no clinical improvement, with a worsening of inflammatory markers and negative blood cultures. On day seven of his hospitalization, a contrast-enhanced lumbosacral MRI scan was repeated, confirming the previous findings: bilateral psoas involvement and small abscess collections (Figure 1), which had no surgical indication according to the Neurosurgery team.

Given a fluctuating clinical and laboratory course, marked by intermittent improvement and subsequent worsening, ceftriaxone was replaced after 11 days with clindamycin for seven days, while flucloxacillin was continued to complete 19 days. Repeat CT imaging was discussed with neuroradiology but deferred in view of the transient clinical improvement and stable neurological examination.

Despite transient clinical improvement, the patient developed recurrent fever and worsening inflammatory markers. Accordingly, on day 19 after admission, when CRP and ESR reached their peak values (97 mg/L and 66 mm/h, respectively), antibiotic therapy was switched to linezolid and ceftriaxone, targeting a possible MRSA etiology underlying the inadequate response to previous treatment.

At that time, repeat CT imaging was planned but subsequently deferred due to clinical and laboratory improvement, including apyrexia, pain relief, and decreasing inflammatory markers (CRP 39 mg/L, ESR 51 mm/h).

**Figure 2:**
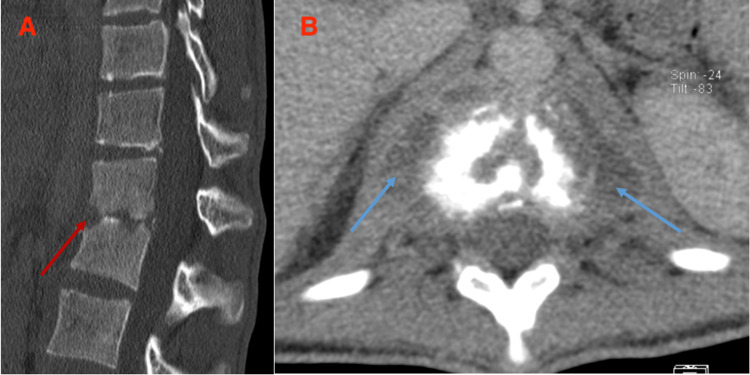
CT sagittal slice, bone window (Image A): destruction of the disc with erosion of the cortical bone (red arrow). CT axial slice, soft-tissue window (Image B): abscess collections (blue arrows).

Imaging was ultimately repeated only one month after admission, following renewed clinical deterioration. At that time, a lumbar CT scan revealed destruction of the intervertebral disc and irregularity of the vertebral endplates at D12-L1, along with alteration of the normal spinal curvature (Figure 2). The size of the bilateral psoas muscle abscesses had also increased, with abscess collections now measuring 27 × 16 × 28 mm on the right and 33 × 15 × 27 mm on the left.

Because of the progressive worsening, the patient was transferred to a tertiary hospital for further management. Thirty-two days after hospital admission, a CT-guided biopsy of the abscess was performed, and bacteriological examination yielded *Pseudomonas aeruginosa*. The antibiotic therapy was subsequently changed to piperacillin-tazobactam and amikacin, which was later switched to gentamicin based on susceptibility testing results.

All other investigations were unremarkable (including those for *Brucella*, *K. kingae*, and *M. tuberculosis*).

**Figure 3:**
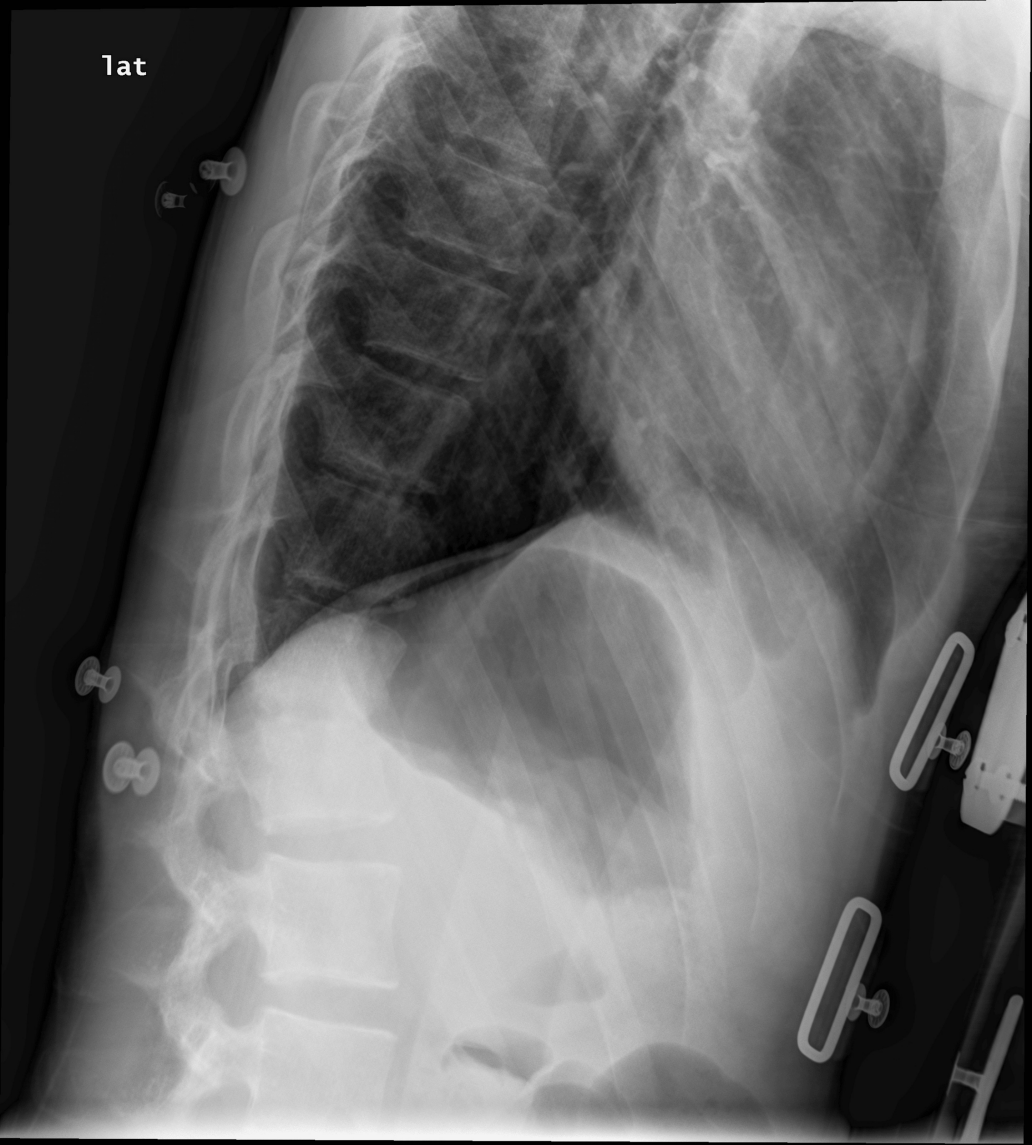
Vertebral destruction with progression of thoracolumbar kyphosis, prompting the need for fixation.

Pain management was optimized by adding gabapentin. Lumbar radiography revealed progression of thoracolumbar kyphosis (Figure 3), and the patient underwent percutaneous posterior fixation from D10 to L2 on the 47th day after admission, which was uneventful. He was then transferred back to the original local hospital to complete his intravenous piperacillin-tazobactam therapy.

He underwent physiotherapy involving muscle reconditioning and gait training, which resulted in progressive functional improvement. He completed a 28-day course of piperacillin-tazobactam. He then completed a two-week course of oral levofloxacin. His clinical course was favorable, and he was discharged two months after admission. He maintained physiotherapy for six months, with good improvements and no limitations in mobility. He has otherwise been well and has had no comorbidities.

## Discussion

We report a rare case of PSD with bilateral psoas abscesses caused by *P. aeruginosa*, highlighting the diagnostic and therapeutic challenges of unusual spinal infections in otherwise healthy adolescents.

The diagnosis of PSD is frequently challenging and delayed due to the non-specific and variable nature of its clinical presentation [1,5]. The initial symptoms are often non-specific and may include back or abdominal pain, reluctance to walk, and low-grade fever. In younger or non-verbal children, the inability to describe or localize pain can impede its recognition, and in infants, symptoms may be limited to irritability, refusal to sit, poor feeding, or general malaise [1,2,7].

Consequently, the average reported diagnostic delay in the literature is approximately 4.8 weeks, though there is significant variation, ranging from as little as two days to as much as 60 weeks [2,5,6]. This delay in diagnosis can significantly contribute to the development of complications, including abscess formation, vertebral destruction, and spinal deformity [4]. Therefore, maintaining a high level of suspicion is essential when evaluating children with unexplained gait disturbances, back pain, or refusal to bear weight, particularly if symptoms persist or worsen over time.

*P. aeruginosa* is an uncommon cause of SD in adults, accounting for up to 6% of cases. It is classically associated with predisposing factors such as diabetes mellitus, recent spinal surgery or wounds, immunosuppressive therapy, and intravenous drug use [8,9]. Ueda et al. [10] reported a case of an 80-year-old male with multiple comorbidities who developed a *P. aeruginosa*-related iliopsoas abscess and SD.

A review of the literature revealed only two reported cases of PSD caused by *P. aeruginosa*: AlTarayra et al. [11] described a case of lumbar SD resulting from contiguous spread following perforated appendicitis in an otherwise healthy adolescent. Mazza et al. [12] reported a case of a 15-year-old boy undergoing chemotherapy for medulloblastoma who developed back pain and was diagnosed with T11-T12 and L1-L2 SD. A CT-guided fine-needle aspiration biopsy yielded *P. aeruginosa*. Both of these patients presented similarly with back pain and increased inflammatory markers. Unlike these cases, which had identifiable predisposing factors, our patient was previously healthy with no history of immunosuppression, prior interventions, or hospitalization. The only potential predisposing factor was his regular swimming practice. This could have played a role, despite the absence of skin wounds or other identifiable routes of inoculation.

Several retrospective and cohort studies [1,2,5-7,13,14] conducted worldwide, encompassing more than 400 pediatric SD cases, reported no isolation of *Pseudomonas* species from any patient. Sex was approximately equally distributed between boys and girls, and MRI was the study of choice across all studies. The most frequently identified pathogens across these studies were *S. aureus* and *K. kingae*; however, a substantial proportion of patients had negative microbiological results. The 2023 systematic review by Lashkarbolouk et al. [2] reported that fewer than 30% of children had positive blood or biopsy cultures, with most series showing culture negativity even after invasive sampling. This has been attributed to prior empirical antibiotic administration before sampling, technical limitations in obtaining adequate deep-tissue specimens, and the fastidious growth requirements of certain pediatric pathogens, such as *K. kingae*. These findings support the increasing use of molecular techniques, such as PCR and 16S rRNA sequencing, to enhance pathogen detection and guide targeted therapy [1,2,4,6].

Antibiotic therapy remains the primary treatment for PSD, and prompt initiation is essential to prevent chronic infections, spinal deformities, and neurological complications. Empirical regimens should primarily provide coverage against* S. aureus*, which is the most common causative pathogen across all pediatric age groups [2,6]. Additional consideration should be given to *K. kingae* in children under four years of age, and to MRSA where regionally relevant [2,6]. The most commonly used regimens included third‑generation cephalosporins (especially ceftriaxone/cefotaxime), antistaphylococcal penicillins, and vancomycin, sometimes combined with aminoglycosides or rifampin in severe cases. Once microbiological identification has been obtained, therapy should be adjusted to a targeted regimen guided by the results of susceptibility testing. Although there are no standardized pediatric-specific guidelines, most reviews recommend an initial intravenous course lasting two to four weeks, followed by an oral continuation phase, for a total treatment duration of six to eight weeks, depending on clinical, laboratory, and imaging responses [1,5]. Prolonged therapy may be required in cases of slow clinical response, abscess formation, or infection by atypical organisms [4,6]. The absence of microbiological isolates is a factor associated with poor prognosis [15].

In this case, the absence of microbiological isolates delayed the diagnosis, as evidenced by the slow response to empirical therapy. Once *P. aeruginosa* was identified through a CT-guided abscess biopsy, targeted antipseudomonal therapy led to a rapid improvement in the patient's condition. Recommended agents for *P. aeruginosa* include antipseudomonal β-lactams such as ceftazidime, cefepime, and piperacillin-tazobactam, as well as carbapenems. Newer combinations such as ceftazidime-avibactam and ceftolozane-tazobactam are reserved for resistant strains [9,16,17]. The typical treatment duration ranges from six to eight weeks, although longer courses may be necessary.

For our patient, a CT-guided biopsy was performed as part of the diagnostic workup, allowing definitive microbiological identification and the initiation of targeted antimicrobial therapy. Clinical improvement was observed only after this intervention, underscoring the importance of obtaining a precise etiological diagnosis. Such invasive procedures are generally reserved for cases with a lack of clinical improvement despite appropriate empirical antibiotic therapy, suspected infection by atypical pathogens, or imaging findings of vertebral osteomyelitis suggestive of a neoplastic process [1,4,5].

Despite the fact that the use of antibiotics before puncture will typically reduce the positive rate of biopsy culture, which our patient had already undergone for several weeks, the successful identification of the pathogen may be strongly attributed to the specific target of the biopsy. The literature reports that sampling soft tissues, such as a paravertebral abscess, is associated with a significantly higher positive culture rate than sampling bone tissue; studies have reported abscess culture positivity rates ranging from 43% to 80% [18].

Surgical intervention is reported in fewer than a third of PSD studies and is reserved for complicated cases; overall, it was required in fewer than 15% of cases, whereas in certain studies the proportion reached as high as 52% [1,2,6,7,13]. Key indications for surgery include documented abscesses (e.g., psoas abscess), neurological deficits, and significant mechanical derangement due to spinal instability or severe vertebral body destruction (e.g., kyphosis or misalignment) [6,14]. Complications associated with PSD are reported in 15-20% of cases [1]. In our patient, the progression of vertebral destruction and kyphosis necessitated surgical stabilization, resulting in a good functional outcome.

## Conclusions

This case highlights the diagnostic and therapeutic challenges of PSD, especially when it is caused by unusual or unexpected pathogens, such as *P. aeruginosa*. As the initial symptoms are often subtle and non-specific, diagnosis is often delayed until significant structural damage has occurred. This highlights the importance of early clinical suspicion and prompt imaging evaluation.

If standard empirical therapy fails, an early image-guided biopsy is essential to establish an etiological diagnosis and guide targeted antimicrobial therapy, thereby significantly improving outcomes. Timely diagnosis, personalized treatment, and multidisciplinary management are essential for achieving full recovery and preventing long-term complications.

## References

[1] Yagdiran A, Meyer-Schwickerath C, Wolpers R (2022). What do we know about spondylodiscitis in children? A retrospective study. Children (Basel).

[2] Lashkarbolouk N, Mazandarani M, Ilharreborde B, Nabian MH (2023). Understanding the management of pediatric spondylodiscitis based on existing literature; a systematic review. BMC Pediatr.

[3] Golchoub G, Hosseini I, Alamdari A (2025). Clinical and microbiological profile of spondylodiscitis: a retrospective analysis. BMC Musculoskelet Disord.

[4] Principi N, Esposito S (2016). Infectious discitis and spondylodiscitis in children. Int J Mol Sci.

[5] Afshari FT, Rodrigues D, Bhat M, Solanki GA, Walsh AR, Lo WB (2020). Paediatric spondylodiscitis: a 10-year single institution experience in management and clinical outcomes. Childs Nerv Syst.

[6] Musso P, Parigi S, Bossi G, Marseglia GL, Galli L, Chiappini E (2021). Epidemiology and management of acute hematogenous osteomyelitis, neonatal osteomyelitis and spondylodiscitis in a third level paediatric center. Children (Basel).

[7] Cavalieri S, Pessina B, Indolfi G, Galli L, Trapani S (2022). Spondylodiscitis in pediatric age: a retrospective cohort study. Pediatr Infect Dis J.

[8] Bourghli A, Boissiere L, Obeid I (2019). Thoracic kyphotic deformity secondary to old Pseudomonas aeruginosa spondylodiscitis in an immunocompromised patient with persistent infection foci-a case report. Int J Spine Surg.

[9] Danda GJ, Franco AC, Gomes EA, Montanaro VV, Martins BJ, Viana Bonan de Aguiar V (2023). Carbapenem-resistant Pseudomonas aeruginosa spondylodiscitis treated with ceftazidime-avibactam: a case report with literature review. Infect Drug Resist.

[10] Ueda K, Hayashi K, Azuma SI, Hayashi M (2024). Iliopsoas abscess related to Pseudomonas aeruginosa: a case report. Cureus.

[11] AlTarayra M, Abuzaina KN, Hassouneh AW, Aljabarein OY (2024). Spondylodiscitis following perforated acute appendicitis in a 14-year-old female: a case report. Int J Surg Case Rep.

[12] Mazza E, Spreafico F, Cefalo G, Scaramuzza D, Massimino M (2004). Case report: Pseudomonas aeruginosa-related intervertebral discitis in a young boy with medulloblastoma. J Neurooncol.

[13] Roversi M, Mirra G, Musolino A (2021). Spondylodiscitis in children: a retrospective study and comparison with non-vertebral osteomyelitis. Front Pediatr.

[14] Kang HM, Choi EH, Lee HJ (2016). The etiology, clinical presentation and long-term outcome of spondylodiscitis in children. Pediatr Infect Dis J.

[15] Pola E, Taccari F, Autore G (2018). Multidisciplinary management of pyogenic spondylodiscitis: epidemiological and clinical features, prognostic factors and long-term outcomes in 207 patients. Eur Spine J.

[16] Rempenault C, Pagis V, Noussair L (2021). Treatment of bone and joint infections by ceftazidime/avibactam and ceftolozane/tazobactam: a cohort study. J Glob Antimicrob Resist.

[17] Petersen EK, Hanberg P, Knudsen M (2022). Intermittent short-term infusion vs. continuous infusion of piperacillin: steady state concentrations in porcine cervical spine tissue evaluated by microdialysis. Antibiotics (Basel).

[18] Tang K, Zhang X, Li Y (2025). The biopsy site is critical for bacterial culture after percutaneous biopsy in patients with pyogenic spondylodiscitis. World Neurosurg.

